# Exploring the Waveform Characteristics of Tidal Breathing Carbon Dioxide, Measured Using the N-Tidal C Device in Different Breathing Conditions (The General Breathing Record Study): Protocol for an Observational, Longitudinal Study

**DOI:** 10.2196/resprot.9767

**Published:** 2018-05-24

**Authors:** Daniel M Neville, Hitasha Rupani, Paul R Kalra, Kayode Adeniji, Matthew Quint, Ruth De Vos, Selina Begum, Mark Mottershaw, Carole Fogg, Thomas L Jones, Eleanor Lanning, Paul Bassett, Anoop J Chauhan

**Affiliations:** ^1^ Portsmouth Hospitals NHS Trust Portsmouth United Kingdom; ^2^ Stats Consultancy Ltd Amersham United Kingdom

**Keywords:** capnography, medical device, diagnosis

## Abstract

**Background:**

In an increasingly comorbid population, there are significant challenges to diagnosing the cause of breathlessness, and once diagnosed, considerable difficulty in detecting deterioration early enough to provide effective intervention. The burden of the breathless patient on the health care economy is substantial, with asthma, chronic heart failure, and pneumonia affecting over 6 million people in the United Kingdom alone. Furthermore, these patients often have more than one contributory factor to their breathlessness symptoms, with conditions such as dysfunctional breathing pattern disorders—an under-recognized component. Current methods of diagnosing and monitoring breathless conditions can be extensive and difficult to perform. As a consequence, home monitoring is poorly complied with. In contrast, capnography (the measurement of tidal breath carbon dioxide) is performed during normal breathing. There is a need for a simple, easy-to-use, personal device that can aid in the diagnosis and monitoring of respiratory and cardiac causes of breathlessness.

**Objective:**

The aim of this study was to explore the use of a new, handheld capnometer (called the N-Tidal C) in different conditions that cause breathlessness. We will study whether the tidal breath carbon dioxide (TBCO_2_) waveform, as measured by the N-Tidal C, has different characteristics in a range of respiratory and cardiac conditions.

**Methods:**

We will perform a longitudinal, observational study of the TBCO_2_ waveform (capnogram) as measured by the N-Tidal C capnometer. Participants with a confirmed diagnosis of asthma, breathing pattern disorders, chronic heart failure, motor neurone disease, pneumonia, as well as volunteers with no history of lung disease will be asked to provide twice daily, 75-second TBCO_2_ collection via the N-Tidal C device for 6 months duration. The collated capnograms will be correlated with the underlying diagnosis and disease state (stable or exacerbation) to determine if there are different TBCO_2_ characteristics that can distinguish different respiratory and cardiac causes of breathlessness.

**Results:**

This study’s recruitment is ongoing. It is anticipated that the results will be available in late 2018.

**Conclusions:**

The General Breathing Record Study will provide an evaluation of the use of capnography as a diagnostic and home-monitoring tool for various diseases.

**Registered Report Identifier:**

RR1-10.2196/9767

## Introduction

### Burden of Breathlessness

Respiratory and cardiac diseases that cause breathlessness are both highly prevalent and major causes of health care utilization across the United Kingdom. Asthma is estimated to affect over 3.5 million people, with 250,000 experiencing severe disease with frequent exacerbations [1]. Heart failure (HF) affects over half a million people, while every year up to 1% of the adult population will suffer from a community-acquired pneumonia [2,3]. Many of these patient groups experience comorbid breathlessness such as breathing pattern disorders (BPD) [4]. The combined burden of these breathless patients on the National Health Service (NHS) is enormous, accounting directly for over £2 billion, with an indirect societal cost (time off work and lost productivity) in excess of £6 billion [5-7]. The prevalence of these diseases is increasing [8,9], and it is often becoming difficult for the clinician to diagnose the cause of a person’s breathlessness and, once diagnosed, considerably difficult for the person to monitor their own disease effectively [10]. Furthermore, patients who are at risk of hypercapnic failure (eg, progressive motor neurone disease, MND) often find it difficult to identify when their condition is deteriorating and when they require more intensive support. The need for noninvasive ventilation in these patients is often only recognized when they are very ill and leads to an increased length of stay in hospital and poorer patient outcomes [11].

### Diagnostic Challenge

Current methods of diagnosing the cause of a person’s breathlessness rely on a combination of symptom history and extensive investigations such as spirometry, peak expiratory flow rates, transthoracic echocardiography, blood tests, and radiology. These can be invasive or are effort or technique dependent, making them difficult to perform and often hard to interpret. Many of these conditions lack a personal monitor that can be used by patients at home, making it hard for patients to self-monitor and engage with their management, resulting in poor medicine adherence [12]. There is a need for a simple, effort-independent tool that can aid the clinician in determining a diagnosis and aid the patient in monitoring their condition. A prompt diagnosis allows timely treatment, and accurately identifying when action needs to be taken empowers the patient and ultimately could improve outcomes.

### Capnography

Capnometers graphically measure carbon dioxide (CO_2_) in exhaled breath and provide clinicians with a noninvasive measure of systemic metabolism, circulation, and ventilation. Capnometers have been a standard of care in general anesthesia for over 30 years and are also widely used in critically unwell patients [13]. Their use is now even recommended to assess adequate cardiopulmonary resuscitation in hospital cardiac arrests [14,15]. However, use is currently limited to these specialist hospital areas in part because of the high cost of capnometers and the requirement for integrated information systems by which data are displayed.

### The Cambridge Respiratory Innovations Limited “N-Tidal C” Capnometer

Cambridge Respiratory Innovations Limited (CRiL), a UK-based company, has developed a novel infrared III-V light-emitting diode capnometer that is intended for use by patients at home to monitor their breathing condition. The N-Tidal C data collector device is a class 1, Conformité Européene marked, handheld device that measures the amount of CO_2_ in the user’s breath during normal tidal breathing.

The device consists of the main unit and a disposable mouthpiece. The device takes approximately 2 min to self-calibrate. The patient then breathes normally through the mouthpiece until the device gives an auditory and visual signal that the reading is complete (75 seconds). The device records the full dataset of exhaled CO_2_ for the duration of use, and the data can be downloaded periodically. Data are analyzed using algorithms developed by CRiL to produce waveforms and can be viewed graphically. It has already been successfully used in a longitudinal clinical study with 30 chronic obstructive pulmonary disease (COPD) patients. This has resulted in the collection of over 2600 75-second breath records containing over 50,000 individual TBCO_2_ waveforms. This research demonstrated a clear differentiation in TBCO_2_ waveforms between stable COPD and during a COPD exacerbation [16]. It has also identified the early changes to TBCO_2_ waveform, indicating an ability to predict COPD exacerbations [16].

We aim to establish a substantial TBCO_2_ waveform database in multiple different conditions that cause breathlessness. This study will determine whether this form of capnography can be used as a tool for diagnosis of various respiratory and cardiac conditions by comparing waveforms in different medical conditions with healthy controls. It will also provide longitudinal information in specific patient groups with chronic disease. This will identify whether this use of capnography, as recorded by the N-tidal C device, can be used as a self-monitoring tool by patients at home.

### Aims and Objectives

#### Coprimary Objectives

The coprimary objectives of this study are to explore the characteristics of the TBCO_2_ waveform, as recorded by the N-tidal C device, which can differentiate between different respiratory and cardiac conditions, and to establish a TBCO_2_ profile for healthy controls.

#### Secondary Objectives

The secondary objectives for this study are as follows:

- To identify within-patient changes in the TBCO_2_ waveform that may predict or detect a deterioration of the underlying disease.
- To establish whether the characteristics of any waveform changes before a clinical deterioration are similar in all patients in the same group.
- To identify waveform features that may help inform a larger disease-specific prospective study in the future.
- To describe the relationship between characteristics of the TBCO_2_ waveform and severity of the primary condition of interest (as measured by disease-specific clinical parameters and symptom questionnaires).
- To compare the use of the TBCO_2_ waveform (and the N-Tidal C device) in monitoring different breathing conditions with traditional methods of monitoring disease control.
- To monitor the safety of the device in regular use by participants over a 6-month period.
- To establish the *ease of use* of capnography measurement as a potential method of disease diagnosis and monitoring in all participants.
- To identify the ranges of TBCO_2_ waveform parameter values (minimum and maximum) for the different disease cohorts.

## Methods

### Overview

A longitudinal, observational study of several cohorts of patients with different medical conditions that result in breathlessness.

### Outcome Measures

#### Primary Outcome

For all conditions, the primary outcome of interest is the twice daily TBCO_2_ waveform as recorded by the N-Tidal C device.

#### Safety Outcomes

Any adverse events (AEs) reported during performing the study procedures and any adverse device effects will be recorded.

#### Patient or Health Care Professional Experience Outcomes

A visual analog scale questionnaire to record the *ease of use* and acceptability of the N-Tidal C device to participants and health care professionals will be used at the completion of the study.

### Study Participants

Participants will be drawn from a range of clinical conditions affecting adults that have symptoms of breathlessness.

### Eligibility Criteria

There are overarching eligibility criteria for all clinical populations, in addition to specific eligibility criteria, detailed below for each clinical population, to ensure that the condition of interest is clearly defined and that the participants will be able to complete the study. These are shown in Textboxes 1 and 2.

Additional condition-specific inclusion criteria (definition of required medical condition) are detailed below.

#### Asthma

##### Inclusion Criteria

The inclusion criteria was as follows:

1. A confirmed clinical diagnosis of asthma for ≥ 6months supported by evidence of *any* of the following:
  - Airflow variability, with a variability in forced expiratory volume in one second (FEV_1_) of >20%, with concomitant evidence of airflow obstruction (FEV_1_/ forced vital capacity ratio <70% on spirometry) recorded at any time;
  - Airway reversibility with an improvement in FEV_1_ by ≥12% or 200 ml after inhalation of 400 μg of salbutamol via a metered dose inhaler and spacer within the preceding 12 months;
  - Airway hyperresponsiveness demonstrated by Methacholine (or similar) challenge testing with a provocative concentration of Methacholine required to cause a 20% reduction in FEV_1_(PC20) of ≤8 mg/ml or equivalent test.
2. Moderate to severe asthma defined as British Thoracic Society stage 3 to 5
3. Exacerbation free for >2 weeks (defined as no increased dose or course of oral corticosteroids or antibiotics)
4. Two or more exacerbations in the previous 12 months with at least one exacerbation within the last 6 months.

**Overarching inclusion criteria.**

- Male or female, aged ≥16 years
- Willing and able to provide written informed consent

**Overarching exclusion criteria.**

- Known other lung, chest wall, neuromuscular, cardiac, or other comorbidity or abnormality that would affect spirometry or other measures of lung function or tidal breath carbon dioxide (TBCO_2_) measurements
- In the opinion of the clinical investigator, the participant would have difficulty completing the study procedures consistently over the course of 6 months

#### Breathing Pattern Disorders

##### Inclusion Criteria

Each participant recruited must have a clinical diagnosis of a BPD by a specialist respiratory physiotherapist.

#### Chronic Heart Failure

##### Inclusion Criteria

Each participant recruited must meet the following inclusion criteria:

1. A confirmed clinical diagnosis of chronic HF with both of the following:
  - A left ventricular ejection fraction <40% on most recent imaging within the last 12 months.
  - New York Heart Association class 2 to 4
2. Either (i) admitted with an acute decompensation of their HF to hospital requiring intravenous diuretics or an increase in diuretic dose from baseline (eg, 40 mg or more furosemide) within the last 6 months or (ii) stable outpatient with N-terminal pro b-type natriuretic peptide (NT-proBNP) >400 ng/mL in sinus rhythm or NT-proBNP >1000 ng/mL in atrial fibrillation.

#### Motor Neurone Disease

##### Inclusion Criteria

Each participant recruited must have a confirmed clinical diagnosis of motor neurone disease.

#### Pneumonia

##### Inclusion Criteria

Each participant recruited must have a confirmed clinical diagnosis of pneumonia supported by evidence of consolidation on a chest X-ray or computed tomography imaging.

#### Healthy Volunteers

##### Inclusion Criteria

The inclusion criteria was as follows:

1. No known history of lung, cardiac, or neuromuscular disease (defined as no current clinical diagnosis of, or be receiving treatment for, a lung, cardiac, or neuromuscular disease)
2. Body mass index ≤40
3. Nonsmoker, or ex-smoker with ≤ 5 pack year history

### Sampling and Sample Size

As this is an observational, proof-of-concept study, there is no formal statistical justification of the sample size, as the purpose of this planned investigation is to gather data on TBCO_2_ waveform using the N‑Tidal C device. If 70 participants use the device 2 times daily over a period of 6 months, the database should include up to 25,000 detailed 75-second respiration records, which will allow time series analysis, comparisons, and correlation with other measures of disease monitoring. This study has been funded by a competitive grant awarded by Innovate UK, and the number of participants recruited into each patient cohort has been restricted by the number of available devices built by CRiL based on the amount of grant funding.

### Study Procedures

#### Recruitment

This study will be conducted at Queen Alexandra Hospital, Portsmouth. Identification of potential participants will be performed in outpatient clinics, inpatient wards, and specialist secondary care clinics in the community depending on the disease cohort. Furthermore, potential participants will also be invited to attend patient information events about the study. These events will be held over 2 hours and introduce the study to potential asthma, BPD, and HF participants identified from clinic lists. At these events, all participants will be given a Participant Information Sheet (PIS), be introduced to the device, and allowed to ask any questions. If interested in the study, they will be given an appointment with the study team for a mutually convenient screening visit. Participants will also be identified at respiratory outpatient clinics and specialist respiratory clinics in the community. In these settings, they will be given PIS and an introduction to the study (verbal and written letter). Potential HF and pneumonia participants will be identified from inpatient wards and outpatient HF services, approached, and given a verbal and written introduction to the study, along with a PIS by a member of the research team. Healthy volunteers will be recruited from hospital staff, with an email sent to all staff (through the research communications team) advertising the study. Potential volunteers will be asked to call or email the study team so that further information (PIS) can be sent via email and an appointment arranged to discuss the study further.

#### Screening and Enrollment

Patients who express an interest and give informed consent will be screened for the study at an assessment visit at a convenient date and time. Participants identified at the patient information events will be given an appointment with the study team at the event. Participants who are inpatients will undertake their first assessment after they have provided informed consent—as an inpatient and before discharge from hospital. Healthy volunteers will be invited to an initial assessment visit at a mutually convenient time.

### Study Assessments

#### Schedule

The N-Tidal C capnometer will be explained to the patients, and they will be trained how to use it. Patients will be asked to use the device at home over a period of 6 months for the asthma, BPD, HF, MND, and healthy control cohorts and a period of 2 months for the pneumonia cohort. Participants will be asked to use the device at home 2 times per day (morning and evening), or up to 6 times per day if they feel that they are starting an exacerbation or deterioration of their condition. Hospitalized inpatients will also use the device 2 times per day as inpatients and will then continue with the monitoring after discharge from hospital. All patients will have a clinical examination and medical history recorded appropriate to their condition.

Additional disease-specific data such as lung or cardiac function tests will be collected at baseline, 2, 4, and 6 months and at point of exacerbation, depending on the underlying condition. The outline of assessments at each visit is detailed in Multimedia Appendix 1

Home monitoring will commence as soon as the screening assessments are complete for patients who are recruited from outpatient clinics or specialist secondary clinics in the community. They will commence the day after discharge from hospital for patients who are recruited as inpatients. Home monitoring will include daily N-Tidal C use and three weekly symptom reporting:

1. N-Tidal C: patients will use the N‑Tidal C, 2 times daily (morning and evening), and up to 6 times daily if the patient feels unwell or has a deterioration of their disease throughout the home monitoring period until the final outpatient clinic visit.
2. Symptom reporting: asthma, HF, BPD, and healthy volunteer participants will have their disease specific symptoms recorded 3 times a week via an automated telephone service provided by a company called “Message Dynamics.” This service has already been used and accepted by patients in previous trials and uses participants’ responses to preset questions to evaluate their state of health [17]. The participants will record their symptoms by responding to a series of questions using the telephone keypad. Using the responses recorded, Message Dynamics will automatically alert the study team if a participant’s state of health has deteriorated beyond a given threshold. This will prompt a telephone call from a member of the study team to the participant to assess their condition and, if needed, arrange a clinical review to diagnose an exacerbation. If the participant does not answer the initial automated telephone call, there will be no more than two follow-up calls (a maximum of three in total).

At each study assessment, the use of the N-Tidal C device will be reviewed. The study nurse will check that the patient is using the device correctly and will provide additional training if needed. The TBCO_2_ waveform data will be downloaded from the device, and the study nurse will confirm that the mouthpieces have been appropriately replaced at the required time intervals

Any AEs and any adverse device effects will be recorded. If the patient feels that their condition is deteriorating and or they are experiencing an exacerbation at any point throughout the study, they will contact the study team, and, if appropriate, make an appointment to be assessed as soon as mutually convenient. The diagnosis of an exacerbation will be made by the assessing clinician and appropriate treatment started. If the patient has been diagnosed as having an exacerbation of their disease by a different health care professional (eg, general practitioner, GP, or practice nurse), they will be advised to contact the study team as soon as possible to arrange an exacerbation assessment.

### The Study Device: The N-Tidal C Capnometer

#### Description

The N-Tidal C data collector device is a novel infrared data collector capnometer that is intended for use by patients at home. It is a handheld device that measures the amount of CO_2_ in the user’s breath during normal tidal breathing. It is simple to use; instructions for the patient are provided. The device set consists of the following:

- N-Tidal C Device x 1
- Mouthpieces x 6
- AA Batteries x 12
- Instructions for use (Participant’s) x 1
- Quick start guide (Participants’) x 1
- Storage case x 1

The mouthpieces should be changed each month.

The purpose of the device is to detect changes in the patient’s exhaled CO_2_ waveform. However, the purpose of this planned investigation is only to gather data on TBCO_2_ waveforms using the N‑Tidal C in different medical conditions and healthy volunteers; the device will not be used to inform actions or treatment for the patients.

CRiL is registered as a manufacturer of class 1 medical devices with the Medicines and Healthcare products Regulatory Agency. The N-Tidal C data-collector capnometer is the first of these, and a batch of 70 devices have been built to medical device Good Manufacturing Practices to meet the requirements of EN ISO 13485. All of the devices have been tested individually against set performance criteria as required for EN DIN 60601-90:1990, EN 21647:2004. All parts of the device that have contact with the skin, mouth, or lips have been constructed from medical grade plastic to mitigate biocompatibility risks.

#### Supply, Packaging, and Storage

The company has manufactured a batch of 70 N-Tidal C data collector capnometers, and each device will be allocated a unique serial number, allowing full tracking and traceability. The device history record will reference details of all major components and materials used in its manufacture. CRiL will supply sufficient N‑Tidal C devices to the investigative site for each participant to have use of a single device. CRiL will also supply the replacement mouthpieces to the site for use by the study participants. Each participant will be supplied with six mouthpieces. The study site will be supplied with additional devices and mouthpieces for staff training and demonstration and as additional stock. The N‑Tidal C devices and mouthpieces should be stored in a secure location at room temperature (10°C-30°C).

#### Administration

CRiL will provide appropriate training to study staff on the use of the N-Tidal C capnometer before the study commences. Additional support will be made available should it be required. In turn, the study staff will train the patients in the use of the device and will provide further training if needed. The N‑Tidal C is for use in this study only and is not to be used for any other purpose. The investigator or designee will maintain a full record of device accountability. A device accountability log will be maintained detailing the dispensing and return of the study devices and mouthpieces. Used mouthpieces will be returned at the end of the study for inspection for contamination and degradation of performance; suitable containers will be provided to participants for the return of these items.

#### Device Use

The test will be performed as per the CRiL instructions. Participants will use the N Tidal C data collector device twice daily (morning and evening) and up to 6 times per day if the patient feels their condition is deteriorating or they are having an exacerbation. The capnometer recordings will be stored in the device until they are downloaded periodically throughout the study by the study staff. Participants will hold the device and breathe at their normal, relaxed rate of breathing into a disposable mouthpiece for 75 seconds. They will be asked to do this twice a day for the study period (until 6 months for all participants apart from those with pneumonia, who will cease follow-up at 2 months). The mouthpiece should be changed each month. It should not be used in crowded rooms, near a vehicle exhaust, open flames, cigarettes, immediately after drinking a hot beverage or a fizzy drink, or where there is a strong breeze. These conditions may interfere with the data capture of the device.

### Disease Questionnaires

These will be completed only by the relevant cohort.

#### Asthma Control Questionnaire

The Asthma Control Questionnaire is a validated 7-item questionnaire for assessing the level of asthma control over the preceding 7 days. The questionnaire includes five symptom scores, the frequency of rescue bronchodilator use, and a measure of airway caliber (FEV_1_% predicted). Responses are given on a 6-point scale, and the overall score is the mean of the responses (0=totally controlled, 6=severely uncontrolled). Scores over 1.0 are considered indicative of poor control [18].

#### Asthma Quality of Life Questionnaire

The Asthma Quality of Life Questionnaire is a validated 32-item questionnaire that measures the functional problems (physical, emotional, social, and occupational) that are most troublesome to adults with asthma. Patients are asked to think about how they have been over the previous 2 weeks and respond to each of the 32 questions on a 7-point scale (7=not impaired at all, 1=severely impaired). The overall score is the mean of all 32 responses [19,20].

#### Nijmegen Questionnaire (Breathing Pattern Disorder or Vocal Cord Dysfunction Cohort)

The Nijmegen score is derived from a 16-item questionnaire that collects information on symptoms related to hyperventilation, such as shortness of breath, feeling confused, palpitations, and tingling fingers. Answers are based on a 5-point Likert scale describing frequency of symptoms from “never” to “very often.” A score of over 23 out of 64 suggests a possible diagnosis of hyperventilation syndrome, and the questionnaire can be used to assess the severity of the patient’s symptoms during clinical assessments [21].

#### Dyspnoea-12 Questionnaire (Breathing Pattern Disorder or Vocal Cord Dysfunction Cohort)

The Dyspnoea-12 Questionnaire is a 12-item questionnaire that covers different aspects of breathing—eg, “my breath does not go in all the way,” “my breathing makes me feel miserable,” and “I feel short of breath.” The patient is asked to tick the box that best reflects their breathing “these days” on a 4 point Likert scale (none, mild, moderate, and severe) [22].

#### Vocal Cord Dysfunction Questionnaire

The Vocal Cord Dysfunction Questionnaire is a 12-item questionnaire that has been validated in breathless patients with the condition and can differentiate vocal cord dysfunction from asthma. It rates the impact of 12 symptoms on a 5-point Likert scale (total score range: 12-60). It has also been shown to assess response to treatment [23].

#### Pittsburgh Vocal Cord Dysfunction Index Score

The Pittsburgh vocal cord dysfunction Index is a validated, easy to use clinical tool that assigns patients a weighted score based on symptoms of throat tightness (score of 4) and dysphonia (score of 2), the absence of wheezing (score of 2), and the presence of odors as a trigger for symptoms (score of 3). A cutoff of ≥4 has high sensitivity and specificity for the diagnosis of vocal cord dysfunction [24].

#### Kansas City Cardiomyopathy Questionnaire (Heart Failure Cohort)

The Kansas City Cardiomyopathy Questionnaire is a 23-item, self-administered instrument that quantifies physical function, symptoms (frequency, severity, and recent change), social function, self-efficacy and knowledge, and quality of life (QoL). An overall summary score can be derived from the physical function, symptom (frequency and severity), social function, and QoL domains. For each domain, the validity, reproducibility, responsiveness, and interpretability have been independently established. Scores are transformed to a range of 0 to 100, in which higher scores reflect better health status [25].

#### Curb-65

The CURB-65 is a clinical prediction rule that has been validated for predicting mortality in community-acquired pneumonia. The score is an acronym for each of the risk factors measured. Each risk factor scores one point, for a maximum score of 5:

- Confusion of new onset (defined as an AMTS of 8 or less)
- Blood urea nitrogen greater than 7 mmol/l
- Respiratory rate of 30 breaths per minute or greater
- Blood pressure less than 90 mm Hg systolic or diastolic blood pressure 60 mmHg or less
- Age 65 years or older

A higher score indicates greater severity of disease and a higher risk of mortality [3].

#### Epworth Sleepiness Scale (Motor Neurone Disease Cohort)

The Epworth Sleepiness Scale (ESS) is a self-administered questionnaire with eight questions. Respondents are asked to rate, on a 4-point scale (0-3), their usual chances of dozing off or falling asleep while engaged in eight different activities. The ESS score (the sum of 8 item scores, 0-3) can range from 0 to 24. The higher the ESS score, the higher that person’s average sleep propensity in daily life [26].

### Description of Respiratory Tests

#### Fractional Nitric Oxide

Fractional exhaled nitric oxide will be measured using a NIOX MINO device (Aerocrine AB, Solna, Sweden) or equivalent device for measuring exhaled nitric oxide level, as specified by the manufacturer’s instructions and outlined in the American Thoracic Society and European Respiratory Society (ATS and ERS) standards [27]. This includes collection by controlled exhalation at the recommended controlled expiratory flow rate of 50 ml/s for greater than 6 seconds.

#### Spirometry

Spirometry will be conducted using a spirometer conforming to ATS and ERS standards as specified by the manufacturer’s instructions [28]. Participants will inhale rapidly and completely from functional residual capacity, then will exhale in an initial blast of exhalation, and then continued exhalation until the end of the test. FEV_1_(L), FVC (L), FEV_1_/ FVC ratio, FEF_25-75_(% of predicted value), and peak expiratory flow (PEF; L/min) will be recorded. FEV_1_, FVC, and PEF will be documented as both absolute values and as a percentage of the predicted value.

#### Full Body Plethysmography

Full body plethysmography will be performed conforming to ATS and ERS standards to assess static and dynamic lung volumes and airways resistance [29]. The functional residual capacity, residual volume, transfer factor (t*ransfer factor* for carbon monoxide), and transfer coefficient (carbon monoxide transfer coefficient) will be recorded as absolute values and as a percentage of the predicted value.

#### Arterial Blood Gas

Arterial blood gases (pH, FiO_2_, pO_2_, pCO_2_, BE, HCO_3_, SaO_2_, and SPO_2_) will be measured and recorded at every assessment visit for the MND cohort. If any additional assessments are taken as part of routine care while the patient is hospitalized or in the community, the details will be recorded on the case report form (CRF). For patients who are in hospital, arterial blood gas measurements will be performed using the hospital’s standard equipment.

#### Chest X-Ray

A diagnosis of pneumonia will be made by either a respiratory physician or a radiologist on confirmation of consolidation on a chest X-ray for the pneumonia group. The location of the pneumonia will be recorded on the CRF.

#### Noninvasive Ventilator and Cough Assist Data

Only participants in the MND cohort who are established on home noninvasive ventilation will have their home ventilator data recorded on the CRF at their routine 3-monthly clinic appointment. This will include inspiratory positive airways pressure, expiratory positive airways pressure, average tidal volumes, hours of use of noninvasive ventilation, mask leak, and type of interface used (nasal or full face mask). These data will be taken off the ventilator and, if applicable, cough assist machine, and therefore will not include any additional testing for participants.

### Description of Cardiac Function Tests

#### Transthoracic 2D Echocardiography

An echocardiogram is a test that uses ultrasound waves to measure the function and structure of the heart, including the heart valves and pressures within different chambers. It is part of routine care for patients admitted with an acute decompensation of their HF [30]. A comprehensive echo will be performed in accordance with the British Society of Echocardiography guidelines [31].

#### N-Terminal Pro-B-Type Natriuretic Peptide

The NT pro-BNP blood test will be performed on all patients in the HF cohort. This is a validated diagnostic and prognostic test for people presenting with HF and its assessment is part of the National Institute of Clinical Excellence guidelines [30].

#### New York Heart Association Class

The New York Heart Association classification is a routinely used functional assessment to classify the severity of HF purely based on assessment of impact of symptoms on daily activities. It places patients into one of four categories from class 1 (no limitation of physical activity) to class 4 (symptoms of HF at rest) [32]

### Other Clinical Tests

#### Full Blood Count and Peripheral Blood Eosinophil Count

The full blood count will be taken and recorded in the asthma, HF, and pneumonia groups. In hospital, if further blood samples for routine hematological and biochemistry are taken, then these will be recorded in the CRF.

#### Skin Prick Testing

Asthma participants will have a skin prick test (SPT) performed to determine their atopic status. If the participant has had a SPT performed and recorded within the last 3 years, then this result will be used. A SPT is a simple and safe method of testing a person to determine whether or not they have an IgE-mediated allergic response to common inhaled allergens. SPT’s will be performed by trained and experienced respiratory health care professionals, who are also trained in resuscitation techniques. Five common aero-allergens will be tested for: grass, house dust mite, aspergillus, and cat and dog dander. Atopic status will be demonstrated by a positive SPT (wheal diameter ≥3 mm larger than the negative control) [33].

### Symptom Questionnaires (To Be Reported Three Times a Week via the Automated “Message Dynamics” System)

#### Asthma Cohort Symptoms

Asthma patients will report their symptoms 3 times a week (Monday, Wednesday, and Friday) via the automated telephone system provided by Message Dynamics. Each participant will be telephoned at a predetermined, agreed time of day. If the participant does not answer the initial telephone call, they will receive no more than two follow-up calls (maximum three in total). The presence or absence of four asthma symptoms during a 24-hour period will be recorded (scoring 0 for absence of symptoms or 1 for presence of symptoms) alongside the number of times asthma reliever medication is used. This will provide a symptom score (from 0-4) and combined with the frequency of reliever medication used, a composite score (from 0-6, Table 1). Questions asked on the automated telephone system will be as follows:

1. Have you experienced regular wheeze in the last 24 hours? (yes or no)
2. Have you been woken at night by your asthma in the last 24 hours? (yes or no)
3. Has your asthma caused chest tightness in the last 24 hours? (yes or no)
4. Have you been more breathless than normal over the last 24 hours? (yes or no)
5. How many times have you used your salbutamol (ventolin) inhaler in the last 24 hours? (option 1: ≤2, option 2: 3-9, and option 3: ≥10).

A score ≥4 would alert the study team and prompt a member of the study team to call the participant.

#### Healthy Volunteer Cohort Respiratory Symptoms

The healthy cohort will be asked to record upper and lower respiratory tract symptoms 3 times a week via the automated telephone system provided by Message Dynamics. Each participant will be telephoned at a predetermined, agreed time of day. If the participant does not answer the initial telephone call, they will receive no more than two follow-up calls (maximum three in total). The presence or absence of three symptoms during a 24-hour period will be recorded (scoring 0 for absence of symptoms or 1 for presence of symptoms). This will provide a symptom score (range 0-3, Table 2). Questions asked on the automated telephone system will be as follows:

1. Have you had a runny or blocked nose in the last 24 hours? (yes or no)
2. Have you had any sinus pain in the last 24 hours? (yes or no)
3. Have you had a cough in the last 24 hours? (yes or no)

#### Breathing Pattern Disorders Cohort Symptoms

The BPD cohort will be asked to record presence of symptoms 3 times a week via the automated telephone system provided by Message Dynamics. Each participant will be telephoned at a predetermined, agreed time of day. If the participant does not answer the initial telephone call, they will receive no more than two follow-up calls (maximum three in total). The presence or absence of three symptoms during the previous 24-hour period will be recorded (scoring 0 for absence of symptoms, or 1 for presence of symptoms). This will provide a daily symptom score (range 0-3, Table 3). Questions asked on the automated telephone system will be as follows:

1. Have you had breathlessness in the last 24 hours? (yes or no)
2. Have you had chest tightness in the last 24 hours (yes or no)
3. Does your breathing feel tense in the last 24 hours? (yes or no)

**Table 1:** Symptoms present in the last 24 hours, reliever use, and assigned score.

Parameter		Score
Wheeze		1
Night waking		1
Chest tightness		1
Breathlessness		1
Maximum symptom score		4
**Reliever use**		
	≤2	0
	3-9	1
	≥10	2
Maximum composite score total		6

**Table 2:** Upper respiratory and chest symptoms present in the last 24 hours and assigned score.

Parameter	Score
Runny or blocked nose	1
Sinus pain	1
Cough	1
Maximum score	3

**Table 3:** Symptoms present in the last 24 hours and assigned score.

Parameter	Score
Breathlessness	1
Chest tightness	1
Feeling tense	1
Maximum score	3

**Table 4:** Symptoms present in the last 24 hours and assigned score.

Parameter	Score
Breathlessness	1
Chest tightness	1
Fatigue	1
Leg swelling	1
Maximum score	4

#### Chronic Heart Failure Cohort Symptoms

The HF cohort will be asked to record symptoms 3 times a week via the automated telephone system provided by Message Dynamics. Each participant will be telephoned at a predetermined, agreed time of day. If the participant does not answer the initial telephone call, they will receive no more than two follow-up calls (maximum three in total). The presence or absence of four symptoms during the previous 24-hour period will be recorded (scoring 0 for absence of symptoms or 1 for presence of symptoms). This will provide a daily symptom score (range 0-4, Table 4). Questions asked on the automated telephone system will be as follows:

1. Have you had breathlessness in the last 24 hours? (yes or no)
2. Have you had chest tightness in the last 24 hours (yes or no)
3. Have you felt more tired than normal in the last 24 hours? (yes or no)
4. Have your legs swelled up more than normal in the last 24 hours? (yes or no)

A score ≥4 would alert the study team and prompt a member of the study team to call the participant.

#### Ease of Use Questionnaire for Participants

A brief self-completed visual analog scale questionnaire will be used to evaluate participants’ opinions of the N-Tidal C device at the end of the study.

#### Test Acceptability Questionnaire for Staff

At the end of the study, a questionnaire will be used to evaluate health care professionals’ opinions of the different study assessments. Informed consent will be obtained from each health care professional to participate within the study. Only health care professionals who taught and assessed the N-Tidal C device during the study to a minimum of 5 patients will be asked to participate.

### Safety Assessment

#### Definitions

##### Adverse Event

An AE is any untoward medical occurrence in a participant taking part in a clinical trial that does not necessarily have to have a causal relationship with the device under investigation. An AE can therefore be any unfavorable or unintended sign, symptom, or disease temporarily associated with the use of the device, whether or not this has a causal relationship with the device under investigation.

##### Adverse Device Event

Adverse device effects are all untoward and unintended medical occurrences in response to a medical device. All cases judged by either a medically qualified professional or the sponsor as having a reasonable suspected causal relationship to the device qualify as a device effect. This also includes any event resulting from insufficiencies or inadequacies in the instruction for use or deployment of the device and includes any event that is a result of a user error.

##### Serious Adverse Events

A serious adverse event is any untoward medical occurrence that

- Results in death
- Is life-threatening. The term “life-threatening” in the definition of “serious” refers to an event in which the participant was at risk of death at the time of the event; it does not refer to an event that hypothetically might have caused death if it were more severe.

- Requires inpatient hospitalization or prolongation of existing hospitalization
- Results in persistent or significant disability or incapacity
- Is a congenital anomaly or birth defect
- Results in other important medical events

Other events that may not result in death, are not life-threatening, or do not require hospitalization may be considered a serious AE when, based upon appropriate medical judgment, the event may jeopardize the participant and may require medical or surgical intervention to prevent one of the outcomes listed above.

##### Serious Adverse Device Effect

A serious adverse device effect (SADE) in a participant taking part in a clinical trial does not necessarily have to have a causal relationship with the device under investigation. However, a SADE is defined as any untoward medical occurrence seen in a patient that can be attributed wholly or partly to the device, which resulted in any of the characteristics or lead to the characteristics of a SADE.

A SADE is also any event that may have led to these consequences if suitable action had not been taken or an intervention had not been made or if circumstances had been less opportune. A SADE will be documented on a serious adverse event form.

Planned admission to hospital for a preexisting condition will not be considered an serious adverse event.

#### Recording and Reporting of Adverse Events

Participants will be asked about the occurrence of any AEs at each follow-up visit and will be asked to report AEs to their local study team between visits. AEs will be assessed by the principal investigator (PI) for causality, intensity, seriousness, and expectedness. Only AEs that have a reasonable possibility of being attributable to the device and any other AE considered to be of clinical significance by the PI as causing harm to the patient will be recorded in the CRF and reported to the sponsor as per their guidelines. Any AEs that do occur and are considered by the PI to be related to the device will be expedited to the sponsor, research ethics committee (REC), and the device manufacturer within 7 days. Lists of the AEs will be provided to the sponsor when requested.

#### Expected Adverse Events and Serious Adverse Events Exempt From Recording

In the different cohorts under investigation, participants may experience an expected deterioration in their condition and a number of serious or nonserious events throughout the course of the study. In the asthma group, participants may be expected to experience:

- An increase in rescue medication usage
- Additional courses of steroids for asthma exacerbations
- Increased unscheduled healthcare usage, including GP and emergency department (ED) visits for deteriorations in asthma control
- Time off work, college, or university because of worsening asthma control
- Hospitalization because of asthma exacerbation

In the HF group, participants may be expected to experience:

- Increase in their diuretic treatment
- Increased unscheduled healthcare usage, including GP and ED visits for deteriorations in their HF symptoms
- Further hospitalizations for acute decompensation of their heart failure.

In the MND group, participants may be expected to experience:

- An increase in medication use
- Additional courses of antibiotics for infections
- Increased unscheduled health care usage, including GP, ED. and high care visits for deteriorations in their MND
- Hospitalization because of worsening respiratory failure
- Natural progression of their MND leading to death.

In the BPD group, participants may be expected to experience:

- Increased unscheduled health care usage, including GP and ED visits for deteriorations in their symptoms
- An increase in any rescue medication use

### Data Handling and Analysis

#### Data Collection and Management

Enrollment into the study will be documented in each participant’s medical notes.

Data collection will comprise

- Paper CRF, including participant characteristics; disease severity; medication lists; clinical examination; and pulmonary, cardiac, radiological, and blood test results.
- Disease specific symptom, control, and QoL questionnaires.
- Paper self-completed questionnaire for participants.
- The N‑Tidal C data collector recordings will be downloaded directly from the device onto computers supplied by the sponsor.

Data management will be conducted by CRiL. All data management procedures will be completed in accordance with CRiL Standard Operating Procedures. Before data being received in-house, it will be monitored at the investigator site. CRF and other data documentation removed from the investigator site will be tracked by the monitor. When errors in clinical data are discovered during data entry, a query will be created. Queries are created when information is missing or is illegible and needs further clarification. Query forms will be sent to the investigator for completion.

#### Data Analysis

##### Primary Analysis

All participants with recorded TBCO_2_ waveforms will be analyzed. Subgroup analysis will be performed on the different study groups, namely, asthma, BPD, HF, MND, pneumonia, and healthy controls.

##### Analysis of End Points

###### Summary Statistics

Demographics or baseline characteristics of each of the study groups (asthma, BPD, HF, MND, pneumonia, and control) will be produced separately, as well as summaries for all groups combined. Normally, distributed continuous variables will be summarized by the mean and SD, whereas the median and interquartile range will be preferred for non-normally distributed continuous variables.

###### Primary Analysis

To establish our primary objective, CRiL will be using a variety of advanced analytic techniques to isolate the key characteristics in the capnogram that can identify each specific medical condition under investigation and to determine whether there is a difference between the different conditions. The team has developed algorithms to analyze specifics of the TBCO_2_ waveform using modern computing technology. We will use an expert in machine learning to analyze the large dataset this study will produce. Specifically, we will analyze all components of the TBCO_2_ waveform. The TBCO_2_ waveform is made up of successive phases; phase 1 is a latency phase representing baseline inspired gas from the ventilatory dead space. Phase 2 is a rapid upward curving slope representing expired mixed air containing CO_2_. Phase 3 is a plateau phase representing the partial pressure of CO_2_ exchanged at the alveoli. The alpha angle (between phase 2 and phase 3 of the waveform), the peak end tidal CO_2_ level, and the beta angle (between phase 3 and the inspiratory downstroke) will all be recorded. Novel parts of the waveform will also be analyzed as more information is found about the TBCO_2_ waveform throughout the study. The analysis will include looking at the consistency of a participants repetitive breathing pattern, any changes between disease and healthy, and any changes between disease state (stable vs exacerbation).

##### Secondary Analyses

Associations between the recorded capnogram and other disease specific measures of severity, clinical condition (stable, exacerbating), and disease control will be examined.

##### Procedure for Dealing With Missing and Spurious Data

The analysis will include any measured data values, with missing values omitted from the analysis. No imputation of missing data will be performed. The data will be examined for outlying values. Where possible, these will be retained in the data analysis and their influence minimized by a data transformation or a nonparametric approach. If such approaches are not practical, the analysis of the primary outcome will be performed twice, with and without the outlying values.

### Ethics

#### Participant Confidentiality

The study staff will ensure that the participants’ anonymity is maintained. The participants will be identified only by initials and a participant’s identity document number on the CRF and any electronic database. All documents will be stored securely and only accessible by study staff and authorized personnel. The study will comply with the Data Protection Act that requires data to be anonymized as soon as it is practicable to do so.

#### Other Ethical Considerations

The study gained approval form South Central—Berkshire NHS Research Ethics Committee (REC reference 17/SC/0284) on July 17, 2017. The ethics committee reviewed and approved the protocol and all study relevant material such as the informed consent forms and PISs. Any changes to protocol or relevant study documents will be approved by the sponsor. Should an amendment be made that requires REC approval, as defined by REC as a substantial amendment, the changes will not be instituted until the amendment has been reviewed and received approval or favorable opinion from the REC and research and development departments. A protocol amendment intended to eliminate an apparent immediate hazard to participants may be implemented immediately, providing that the REC are notified as soon as possible and an approval is requested. Minor amendments as defined by REC as nonsubstantial amendment may be implemented immediately; and the REC will be informed. All participants will have adequate time to consider participation in the study, as per Good Clinical Practice guidelines [34].

There is a possibility that the study procedures reveal potential new, previously unknown disease pathology. This would be more likely to occur in our healthy controls. If such a circumstance occurs, then the participant will be told of the results and immediately referred to the most appropriate NHS department for further review. With the participant's consent, a letter will be written to their GP explaining the findings.

#### Informed Consent

It is the responsibility of the investigator, or a person designated by the investigator (if acceptable by local regulations), to obtain written informed consent from each person participating in this study, after adequate explanation of the aims, methods, anticipated benefits, and potential hazards of the study using the PIS. The consent process will be documented in the participant’s notes.

The process for obtaining participant informed consent will be in accordance with the REC guidance, and Good Clinical Practice and any other regulatory requirements that might be introduced. The PI or delegate and the participant or other legally authorized representative shall both sign and date the informed consent form before the person can participate in the study. The participant will keep the PIS and a copy of the signed and dated consent form. The original will be retained in the trial master file. A second copy will be filed in the participant’s medical notes and a signed and dated note made in the notes of when the PIS was provided and that informed consent was obtained for the study.

The decision regarding participation in the study is entirely voluntary. The investigator or their nominee shall emphasize to them that consent regarding study participation may be withdrawn at any time without penalty or affecting the quality or quantity of their future medical care, or loss of benefits to which the participant is otherwise entitled.

#### Patient and Public Involvement

Patient and public involvement in this study has been obtained from patients with firsthand experience of living with breathing-related diseases. Initially, patients from our Wessex Asthma Network identified the need for new tools (especially for those with severe disease) to be developed and tested that allows them to reliably monitor their asthma at home or at the surgery or hospital, which does not involve forced breathing, and can alert them when an exacerbation is about to occur. If it could also detect whether someone has asthma or a different cause of their breathlessness, then that would help many more people get an early diagnosis and treatment.

Working with patients in Wessex, we approached several innovators and UK companies to challenge them to produce new tools for people who suffer from conditions that affect their breathing. CRiL is such a company which has developed a unique tool (N-Tidal C) that could be used for exactly this purpose. Working with our patient advisors, we have designed a simple study with this new device that will compare information from patients with different breathing conditions to healthy people.

Our patient representatives have specifically contributed to the study design, screening and recruitment strategies, shape of the protocol, PIS, the lay summary, and the participant self-completion questionnaire. They are also satisfied that this study will not be excessively burdensome to participants.

## Results

Recruitment to the General Breathing Record Study is ongoing. It is anticipated that results will be available by late 2018.

## Discussion

The General Breathing Record Study will provide evaluation of a new handheld capnometer, the N-Tidal C. It will assess the use of capnography in differentiating different respiratory and cardiac diseases compared with healthy controls. It will provide some data on the use of capnography as a tool to detect deterioration in disease state. The study will also allow us to develop the device further in response to participant feedback.

## Appendix

Multimedia Appendix 1 Schedule of visit assessments.

## Abbreviations

AE: adverse event
BPD: breathing pattern disorders
COPD: chronic obstructive pulmonary disease
CRF: case report form
CRiL: Cambridge Respiratory Innovations Limited
ED: emergency department
ESS: Epworth Sleepiness Scale
FEV1: forced expiratory volume in 1 second
FVC: forced vital capacity
HF: heart failure
MND: motor neurone disease
NHS: National Health Service
NT-proBNP: N-terminal pro b-type natriuretic peptide
PEF: peak expiratory flow rate
PI: principal investigator
PIS: Participant Information Sheet
QoL: quality of life
REC: research ethics committee
SADE: serious adverse device effect
SPT: skin prick test
TBCO_2_: tidal breathing carbon dioxide
