# Supplementary material for: Exploring the Waveform Characteristics of Tidal Breathing Carbon Dioxide, Measured Using the N-Tidal C Device in Different Breathing Conditions (The General Breathing Record Study): Protocol for an Observational, Longitudinal Study
Source: JMIR Res Protoc. 2018 May 24;7(5):e140. doi: 10.2196/resprot.9767 (PMC5992452; doi:10.2196/resprot.9767)
Supplement: Multimedia Appendix 1 [file resprot_v7i5e140_app1.pdf]

Condition and assessments	Baseline	2 months	4 months	6 months	At exacerbation
**All Participants**					
Informed Consent	X				
Demographics	X				
Height and Weight	X				
Medical History	X				
Medication Review	X	X	X	X	X
Vital Signs	X	X	X	X	X
N-Tidal C device					
Demonstration and training	X				
Check use of device	X	X	X	X	X
Download device data		X	X	X	X
Collect device				X	
**Asthma**					
Clinical Assessment	X	X	X	X	X
Symptom questionnaires (Asthma Control Questionnaire, Asthma Quality of Life Questionnaire) and asthma symptom score	X	X	X	X	X
Spirometry	X	X	X	X	X
Fractional exhaled Nitric Oxide (FeNO)	X	X	X	X	X
Peak Expiratory Flow Rate	X	X	X	X	X
Peripheral Blood Eosinophil Count	X				X
Skin Prick Test (most recent within 3 years)	X				
Full Body Plethysmography	X				
**Breathing pattern disorders**					
Clinical Assessment	X				
Physiotherapist re-training & review		X	X	X	
Diagnosis Questionnaires (Nijmegen, Pittsburgh Index)	X				
Symptom Questionnaires (Nijmegen, Dyspnoea-12, Vocal Cord Dysfunction Questionnaire)	X	X	X	X	
Symptom diary review	X	X	X	X	X
Spirometry	X				
Full Body Plethysmography	X				
**Heart Failure**					
Clinical Assessment, weight, Blood Pressure & Heart Rate	X	X	X	X	X
Transthoracic Echocardiography	X				X
N-Terminal pro-B type Natriuretic Peptide	X	X	X	X	X
New York Heart Association Class	X	X	X	X	X
Symptom questionnaire (Kansas City Cardiomyopathy Questionnaire)	X	X	X	X	X
**MND**					
Clinical Assessment	X	X	X	X	X
Spirometry (most recent)	X	X	X	X	
PEFR	X	X	X	X	X
Cough Assist Device data (if applicable)	X	X	X	X	X
Arterial Blood Gas	X	X	X	X	X
Ventilator data (if applicable)	X	X	X	X	X
Epworth Score	X	X	X	X	X

**Pneumonia (2 month cohort, extended to 4 months if unresolved)**					
Clinical assessment	X	X	(X)		
Chest X-Ray	X	X	(X)		
Full Blood Count	X				
CURB-65 Score	X				
**Healthy volunteers**					
Clinical Assessment	X			X	
Spirometry	X			X	
Electrocardiography	X				
FeNO	X			X	
Symptom review – upper and lower respiratory tract symptoms	X	X	X	X	
